# An innovative wireless electrochemical card sensor for field-deployable diagnostics of Hepatitis B surface antigen

**DOI:** 10.1038/s41598-023-30340-5

**Published:** 2023-03-02

**Authors:** Prinjaporn Teengam, Pisit Tangkijvanich, Natthaya Chuaypen, Orawon Chailapakul

**Affiliations:** 1 Electrochemistry and Optical Spectroscopy Center of Excellence, Department of Chemistry, Faculty of Science, Chulalongkorn University, Pathumwan, Bangkok, 10330 Thailand; 2 Center of Excellence in Hepatitis and Liver Cancer, Department of Biochemistry, Faculty of Medicine, Chulalongkorn University, Pathumwan, Bangkok, 10330 Thailand

**Keywords:** Biochemistry, Biotechnology, Biomarkers, Chemistry

## Abstract

A wireless-based detection utilizing an innovative electrochemical card (*e*Card) sensor controlled by a smartphone was developed for targeting Hepatitis B surface antigen (HBsAg). A simple label-free electrochemical platform allows a convenient operation for point-of-care diagnosis. A disposable screen-printed carbon electrode was modified straightforwardly layer-by-layer with chitosan followed by glutaraldehyde, allowing a simple but effective, reproducible, and stable method for covalently immobilizing antibodies. The modification and immobilization processes were verified by electrochemical impedance spectroscopy and cyclic voltammetry. The smartphone-based *e*Card sensor was used to quantify HBsAg by measuring the change in current response of the [Fe(CN)_6_]^3-/4-^ redox couple before and after the presence of HBsAg. Under the optimal conditions, the linear calibration curve for HBsAg was found to be 10–100,000 IU/mL with a detection limit of 9.55 IU/mL. The HBsAg *e*Card sensor was successfully applied to detect 500 chronic HBV-infected serum samples with satisfactory results, demonstrating the excellent applicability of this system. The sensitivity and specificity of this sensing platform were found to be 97.75% and 93%, respectively. As illustrated, the proposed *e*Card immunosensor offered a rapid, sensitive, selective, and easy-to-use platform for healthcare providers to rapidly determine the infection status of HBV patients.

## Introduction

Hepatitis B virus (HBV) infection has been associated with high morbidity and mortality in relation to related diseases such as chronic hepatitis, liver cirrhosis, and hepatocellular carcinoma^1^. Despite the availability of vaccines for preventing infection and thus lowering the prevalence rate, HBV remains a major public health problem worldwide, causing hundreds of thousands of deaths each year^2^. Early diagnosis of HBV infection undergoes a series of defined phases that can help clinicians in disease tracing and slow or prevent the spread of the disease. Classical methods for HBV diagnostics, including polymerase chain reaction (PCR)^3^, enzyme-linked immunosorbent assays (ELISA)^4,5^, radioimmunoassay (RIA)^6^, enzyme immunoassay (EIA)^7,8^, electrochemiluminescence immunoassay (ECLIA)^9^ and chemiluminescent microparticle immunoassay (CMIA)^10,11^ have proven sensitive and effective for HBV detection but they currently employ technologies which depend on a laboratory-based system, complex instrument, and specialized expertise or trained technical staff. These factors serve to increase the cost per test, and time between sample collection and result, limiting their utility for routine use at the point of care.

Rapid diagnostic tests (RDTs) for HBV detection could be achieved by Point-of-care testing (POCT). POCT is defined as a system designed for a complete diagnostic platform that drastically reduces sample-to-answer time and improves patient outcomes^12^. This system has been developed to address the challenge in terms of miniaturization and simplification, especially for field deployment. The current format of HBV POCT has mostly relied on lateral flow assays (LFAs) by employing serological assays and the visual detection of antigen–antibody immunocomplex using a nanoparticle label^13,14^. LFAs is typically qualitative (yes/no) or semi-quantitative at best when combined with an external reader. While LFAs serve a valuable purpose in diagnosis fields, they are relatively insensitive since enzymatic amplification for signal detection is not required, giving rise to false negative rates. Therefore, significant improvements in alternative methods for HBV diagnostics still remain to fulfill the demand of sensitive, simple, rapid, and affordable POCT devices.

Electrochemical biosensors have emerged as a promising point-of-care diagnostic platform owing to their reliability, high sensitivity and ability to be designed as a miniaturized form for on-site analysis^13,14^. These performance features are valuable alternatives for the diagnosis of a variety of diseases^15–18^. In a typical electrochemical immunoassay, the electrochemical biosensor is combined with functional proteins immobilized on a transducer surface, and the electrochemical signals are either directly or indirectly transduced from specific biochemical events of the immobilized bio entity with its binding target (e.g., antigen–antibody interaction and/or enzyme–substrate reaction) into a concentration-dependent measurable signal. While direct detection relies on intrinsic electroactivity from labels and/or electroactive indicators^19,20^, indirect process-enabled label-free strategies^21–23^ are favorable for eliminating the complicated detection process, providing rapid sample response, low cost, and high simplicity.

Another key issue that could determine the electrochemical biosensor performance is the quality of the gentle but effective immobilization of recognition biomolecules onto the electrode employed as a transducer for electron movement. A requisite for well-functionalized electrode and nondestructive fixation in terms of biological activity of the biomolecules upon immobilization should be a concern due to inappropriate methods may cause loss of activity and low biocompatibility. Moreover, for the achievement of good sensitivity, high loading of the captured molecules onto the active electrode area is a crucial condition since the surface density of the receptor on the electrode governs the magnitude of the electrochemical signal generated on analyte binding^24–26^. Therefore, the strategy for immobilization of recognition molecules onto electrode must be decided. Among the surface preparations, Chitosan (CHT) has been used as an interface material since it provides outstanding features including ease of modification, biocompatibility, nontoxicity, and being cheaply available in various forms^27,28^. Stable immobilization of the recognition molecules in chitosan matrices on the electrode surface under mild conditions has proved effective. It is also characterized by the availability of a large number of amino and hydroxyl-based functional groups that can excellently serve for the covalent immobilization of biosensing elements by introducing a proper choice of reagent, for instance, glutaraldehyde, allowing the covalent bond formation or affinity conjugation with other functional components^28^. Thus far, chitosan-based electrochemical immunosensors have been applied in various applications according to their obvious performance^29–33^. However, using the chitosan-supported electrochemical biosensor as a handle in point-of-care clinical trials remains a challenge.

Here, we present an innovative diagnostic device for targeting Hepatitis B surface antigen (HBsAg) based on a novel electrochemical sensor architecture designed as a portable card-sized potentiostat. The specific antibody was covalently immobilized onto CHT-modified electrode by employing glutaraldehyde as a cross-linking reagent. Quantification based on the label-free immunoassay by measuring the current response of [Fe(CN)_6_]^3−/4−^ redox solution was performed using amperometric detection. Results will automatically be generated on the smartphone that activates the NFC system, providing real-time diagnostics and surveillance capability. Analytical performance, including sensitivity, cross-reactivity and precision was evaluated. The proposed platform technology was then applied in real clinical samples of patients with chronic HBV infection, demonstrating high sensitivity and specificity and a good correlation with a chemiluminescence microparticle immunoassay (CMIA). Utilizing the simple, portable and rapid sample-to-answer features, our proposed platform could be served as field-deployable diagnostics.

## Results and discussion

### Characterization of modified electrode

In order to confirm the activation and cross-linking of the chitosan-modified electrode with glutaraldehyde, the surface functional groups were characterized by infrared spectroscopy with ATR accessory (ATR-FTIR). The FTIR spectra of three electrodes, including the unmodified screen-printed carbon electrode (SPCE), the chitosan-modified SPCE (CHT/SPCE), and the crosslinked glutaraldehyde (G) on the chitosan-modified electrode (G/CHT/SPCE), were compared in Fig. S1. The characteristic bands of the carbon-based electrode (red line) including C-O stretching at 1025 cm^−1^ and C=O stretching at 1517 cm^−1^ were observed on unmodified SPCE^34^. These bands belong to a carboxylic functional group of esters on the carbon surface. In the presence of CHT, the absorption band of CHT/SPCE (green line) provided the peak of N–H and O–H stretching at 3324 cm^−1^, C–H stretching at 2881 cm^−1^. The bands at 1383 cm^1−^, 1503 cm^−1^ and 1609 cm^−1^ correspond to the CH_3_ bending, C-N stretching and C=O stretching, respectively^35^. Moreover, other peaks at 1073 cm^−1^ and 1119 cm^−1^ attributed to C–OH stretching and C–O–C bending. After the CHT/SPCE was incubated with glutaraldehyde (blue line), the peaks of CHT at 1383 cm^−1^ and 1119 cm^−1^ disappeared, suggesting that these peaks may be hindered by glutaraldehyde crosslinked structure of CHT^35–37^. Indeed, we found the new sharp peak at 1699 cm^−1^ which can be assigned to the C=N stretching in Schiff’s base formed by the cross-link reaction of chitosan and glutaraldehyde^35,38^. This evidence ensures the success of cross-linking reaction between CHT and G.

### Electrochemical characterization

**Figure 1:**
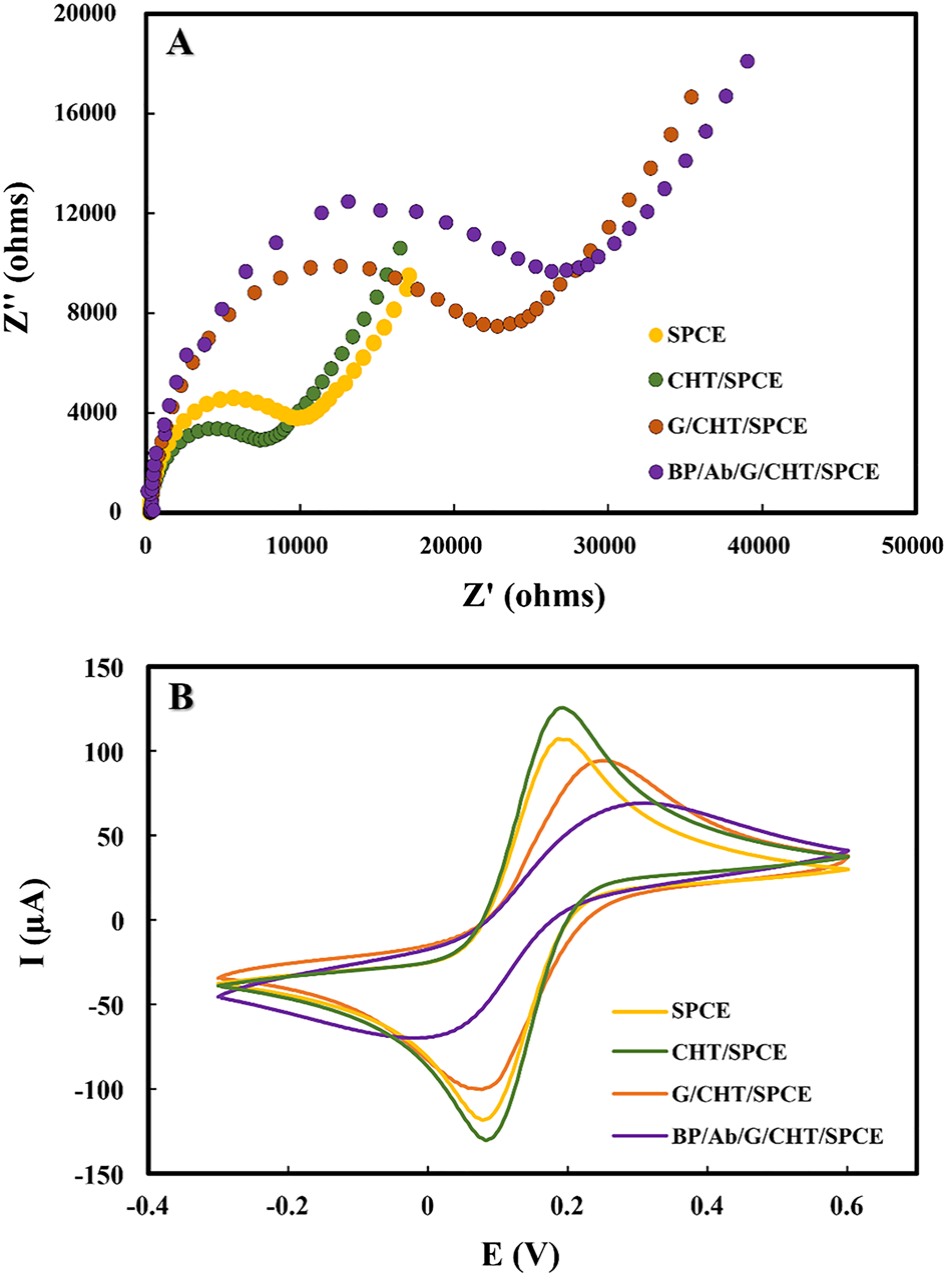
(**A**) Nyquist plots and (**B**) cyclic voltammograms of SPCE, CHT/SPCE, G/CHT/SPCE, and BP/Ab/G/CHT/SPCE using 5 mM [Fe(CN)_6_]^3−/4−^ in 0.1 M KCl.

Electrochemical impedance spectroscopy (EIS) and cyclic voltammetry (CV) were employed to investigate the interfacial electron transfer efficiency for the stepwise electrode modification. The charge transfer resistance (R_ct_) derived from a semicircle diameter of the Nyquist curve from EIS indicates electron transfer capability between the electrode surface and redox reporter (5 mM [Fe(CN)_6_]^3−/4−^). As shown in Fig. 1A, the unmodified SPCE provided a semicircle with an R_ct_ of 11,390 ± 15 Ω. The R_ct_ decreased to 9548 ± 28 Ω after modifying the electrode surface with chitosan (CHT/SPCE), implying the enhancement of electron transfer rates. This is due to the redox probe [Fe(CN)_6_]^3−/4−^ being electrostatically entrapped on the electrode surface by residual amino groups of chitosan prepared at low pH. In contrast, when glutaraldehyde was added, the R_ct_ increased (27,850 ± 26 Ω) due to the formation of chitosan-glutaraldehyde cross-linking, which hampered the electron transfer process. After immobilizing the antibody on the electrode surface, followed by blocking a nonspecific surface with BlockPro™ (BP) (BP/Ab/G/CHT/SPCE), the R_ct_ (31,240 ± 25 Ω) significantly increased due to the antibody's non-electroactive property as well as the blocking agent, which resulted in strong resistance to electron transfer.

The success of electrode modification was further characterized by CV using 5 mM [Fe(CN)_6_]^3−/4−^. The obtained electrochemical behavior at different stages of electrode preparation are correlated with those obtained from EIS. As displayed in Fig. 1B, the anodic peak current (I_pa_) of SPCE was obtained at 102.5 ± 0.8 µA. The increase in I_pa_ to 128.4 ± 1.2 µA was observed after the addition of chitosan (CHT/SPCE). Glutaraldehyde was then cross-linked with chitosan (G/CHT/SPCE), providing the decrease of I_pa_ (91.7 ± 0.19 µA) as the cross-link reaction completely occurred on the electrode surface. Following the antibody immobilization and non-specific surface blocking (BP/Ab/G/CHT/SPCE), the I_pa_ further decreased to 62.8 ± 1.1 µA, indicating the binding of antibody on the surface of G/CHT/SPCE. Additionally, the reduction of I_pa_ was attributed to the non-electronic conductivity and the shielding effect of antibody and blocking agent. These results, therefore, confirmed the success of electrode modification. The histograms of the R_ct_ value and the current signal obtained in a stepwise electrode modification are shown in Fig. S2.

### Amperometric detection of HBsAg using smartphone-based eCard sensor

**Figure 2:**
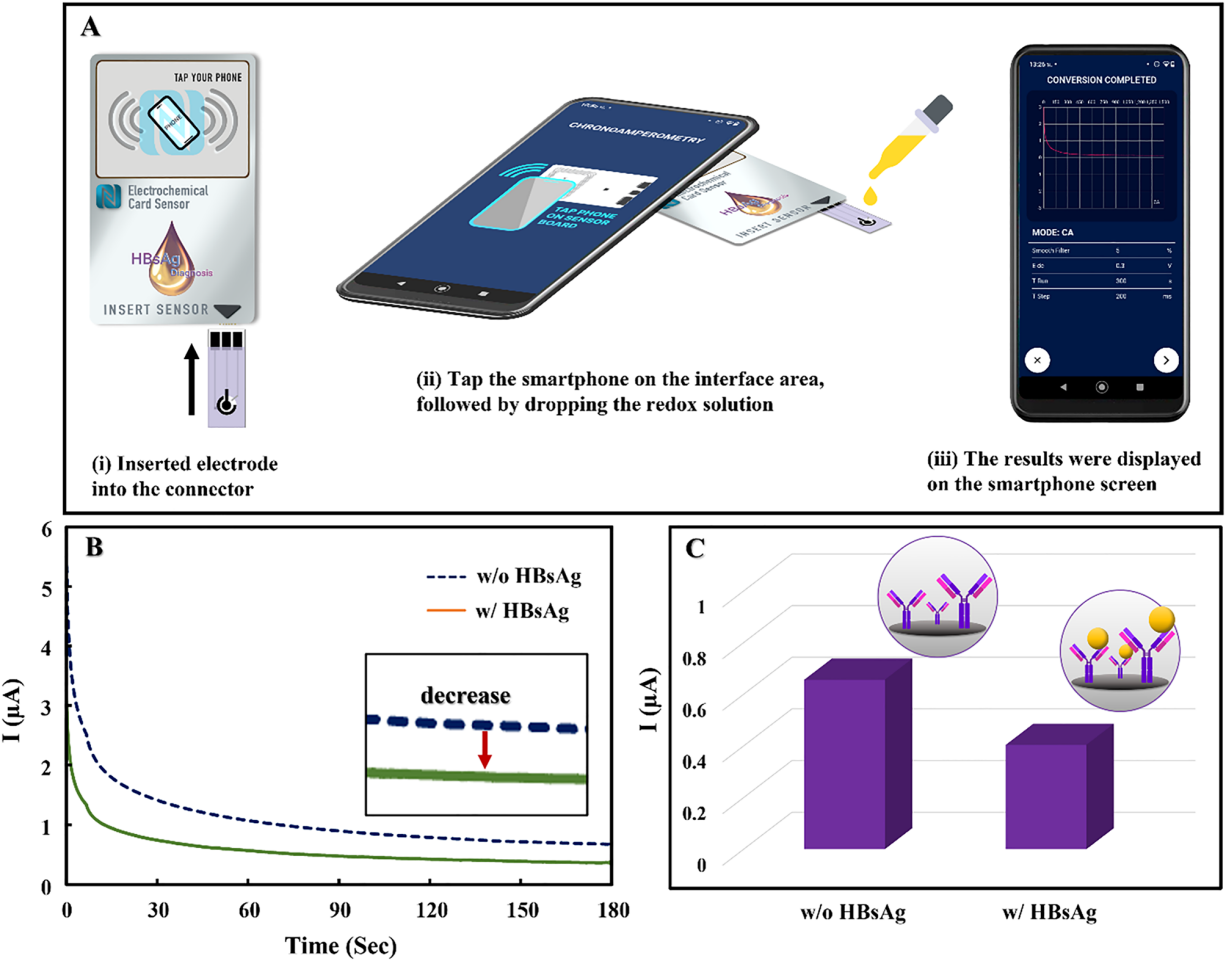
(**A**) The operation procedures of smartphone-based amperometric detection, (**B**) amperometric curve, and (**C**) the current response of BP/Ab/G/CHT/SPCE before and after addition of 100 IU/mL HBsAg using 2 mM [Fe(CN)_6_]^3−/4−^ in 0.1 M KCl.

Herein, the NFC smartphone-based amperometric detection for detecting HBsAg was performed using the innovative portable *e*Card sensor. Figure 2A exhibits the operation procedure of the system. To initiate the electrochemical detection process, the prepared electrode was first inserted into the connector of the *e*Card sensor (i). Next, the electrochemical method and all optimal parameters were set from the Chemister application in the smartphone, followed by tapping the smartphone onto the interface area (ii). After the wireless connection was established, the redox solution of 2 mM [Fe(CN)_6_]^3−/4−^ was added to cover all three electrodes and the detection process began. Finally, the detection result was displayed on the smartphone screen (iii). The amperometric detection before and after addition of HBsAg target (100 IU/mL) was then carried out as displayed in Fig. 2B. The current response of 2 mM [Fe(CN)_6_]^3−/4−^ decreased in the presence of HBsAg (Fig. 2C), confirming the formation of antigen–antibody complex on the electrode surface which hindered the electron transfer of redox solution to the electrode surface. These results clearly indicated the capability of the NFC smartphone-based system for detecting HBsAg.

### Optimization of experimental variables

To achieve the optimal sensing system, relevant parameters including chitosan concentration, glutaraldehyde concentration, antibody concentration, immobilization time and incubation time were optimized. The influence of these variables on the current response of 2 mM [Fe(CN)_6_]^3−/4−^ redox couple was investigated using amperometric detection.

#### Chitosan concentration

Chitosan served as the amino group donor for antibody immobilization. As shown in Fig. S3A, the obtained current signal increased upon the increment of chitosan concentration until it plateaued at a concentration of 0.1% (w/v). Higher chitosan concentrations resulted in the formation of a film interface, which limited mass transport to the electrode surface. Hence, the 0.1% (w/v) of chitosan concentration was selected for the following experiments.

#### Glutaraldehyde concentration

The capture antibody was immobilized on the electrode surface by using glutaraldehyde as a cross-linker to form a covalent bond with chitosan through its aldehyde groups. Accordingly, the impact of glutaraldehyde concentration was then studied. Fig. S3B shows that the current signal decreased with the increasing glutaraldehyde concentration. This is due to the surface of chitosan-modified electrode becoming more hydrophobic when crosslinked with more glutaraldehyde, resulting in lower accessibility of redox solution to electrode surface. Therefore, 1% (v/v) of glutaraldehyde was selected as the optimal condition.

#### Antibody concentration

Next, the influence of antibody concentration was optimized using a serial dilution series of HBsAg monoclonal antibody. As shown in Fig. S3C, the decrease in current signal was observed at higher concentration of antibody and reached a plateaue at a concentration of 1:100 due to a saturation of the electrode surface density which affected the accessibility of more Ab to immobilize the electrode surface. Thus, a concentration of 1:100 was chosen for further studies.

#### Immobilization time

Another factor that can affect the binding activity of antibody on electrode surface is immobilization time. The effect of antibody immobilization time was therefore investigated. It was observed that the current signal decreased with increasing immobilization time (Fig. S3D), indicating higher immobilization efficiency over a longer period and being likely to be constant after 45 min, Accordingly, an immobilization time of 45 min was selected for further experiment.

#### Incubation time

Finally, the influence of incubation time between the target HBsAg and antibody immobilized on the electrode surface was optimized. The HBsAg target (100 IU/mL) was incubated on the electrodes at different times followed by washing with PBS (pH 7.4). As illustrated in Fig. S3E, the change in current response (ΔI) obtained before and after incubation with HBsAg greatly increased with the increase of incubation time and reached a plateaue at 45 min. At longer incubation time, the electrode surface became saturated which limited the mass transport of redox molecules to access the electrode surface^39^. Therefore, the incubation time of 45 min was selected as an optimal condition.

### Analytical performance

**Figure 3:**
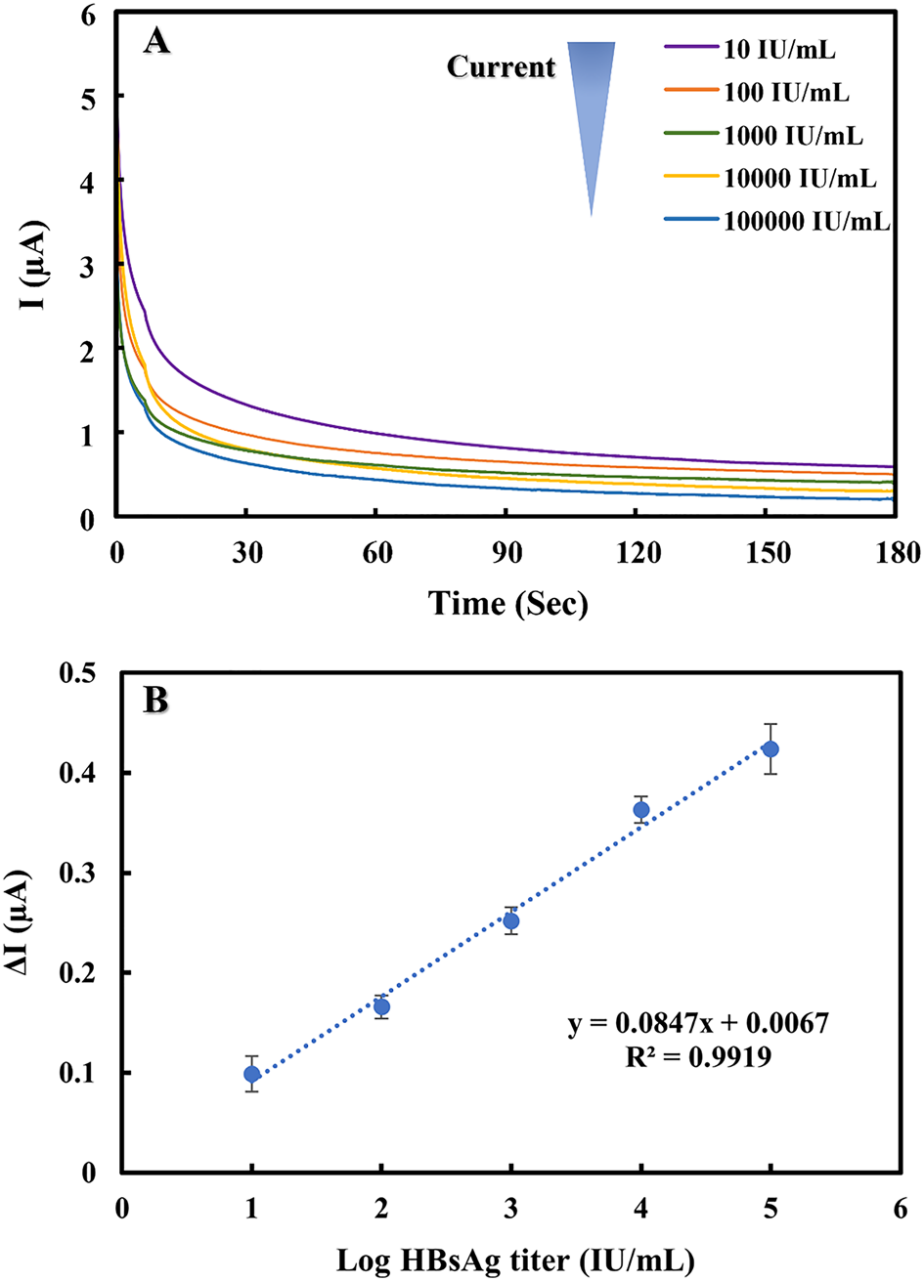
(**A**) Amperometric curve of the proposed immunosensor using 2 mM [Fe(CN)_6_]^3−/4−^ at different concentrations of HBsAg in the range of 10 IU/mL to 100,000 IU/mL. (**B**) The calibration plot of the current change (∆I) as a function of logarithmic HBsAg titer.

The analytical performance of the proposed system was evaluated using a dilution series of HBsAg titers. The smartphone-based real-time monitoring of current response as a function of HBsAg titers was performed. Figure 3A reveals that higher HBsAg titers led to lower current signals. The calibration curve was constructed by plotting between the current change (ΔI) obtained from I_blank_-I_HBsAg_ (where I_blank_ and I_HBsAg_ are obtained before and after incubation with target HBsAg) against logarithmic HBsAg titers as depicted in Fig. 3B. A linearity was observed in the titers range from 10–100,000 IU/mL with a correlation coefficient of 0.9919. The limit of detection (LOD, S/N = 3) was calculated to be 9.55 IU/mL. The analytical performance of the proposed wireless NFC sensing system and the other electrochemical immunosensor used for HBsAg detection is summarized in Table S1.It can be seen that our proposed system can achieve a sufficiently low detection limit for HBsAg detection, ensuring the capability of applying at clinically relevant levels^40,41^. Notably, the NFC *e*Card sensor provided the advantages of portability which can be used for field-deployable diagnosis and the simplicity of use benefiting from the label-free assay which can be easily and inexpensively prepared compared to the other HBsAg immunosensors. Moreover, the results obtained from our NFC *e*Card sensor can be simply operated on smartphone, providing a rapid sample-to-answer diagnostic.

### Cross-reactivity

**Figure 4:**
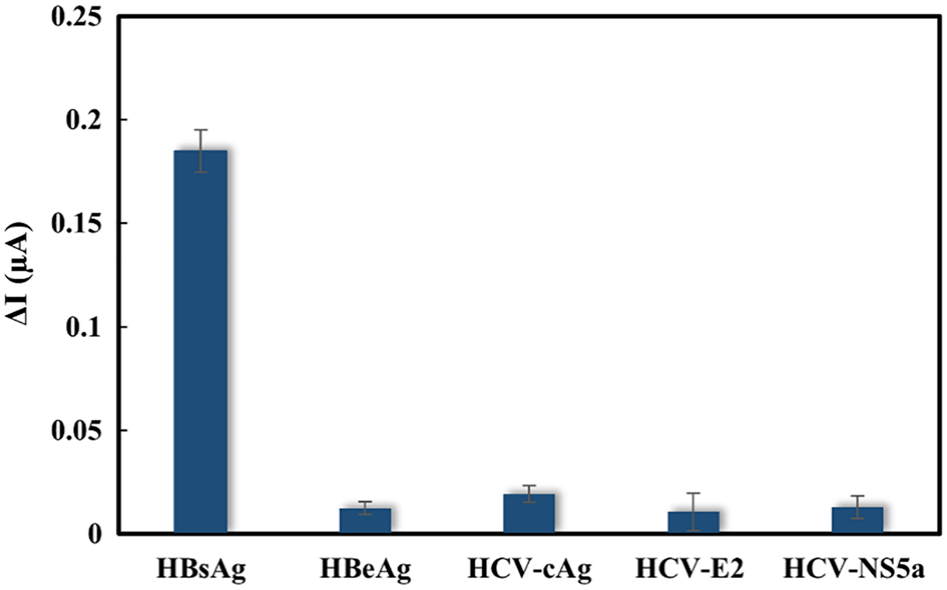
Cross-reactivity of the proposed HBsAg electrochemical immunosensor.

In order to determine the cross-reactivity of various target viruses, the comparison of current response obtained from HBsAg and other viruses including Hepatitis B virus e antigen (HBeAg), hepatitis C virus core antigen (HCV-cAg), HCV-E2 protein, and HCV-NS5a protein was observed. The ampermetric curve derived from the proposed sensor after addition of HBsAg and various viruses antigen was shown in Fig. S4. All interferences were prepared at 10 order of magnitude higher than that concentration of HBsAg. As shown in Fig. 4, a negligible response was observed for all interferences because they provided changes in current response (ΔI) of less than 10%, indicating that the developed sensing platform for HBsAg has good selectivity and specificity.

### Assay stability

For point-of-care diagnostics, a field-deployable sensor should be able to store for a reasonable lifetime without a significant decrease in efficiency. To assess the stability of our biosensing device, the HBsAg immunosensor was prepared and stored at 4 °C before collecting the amperometric current response of 100 IU/mL of HBsAg day after day. As shown in Fig S5, setting the freshly prepared test at 100% efficiency, the %efficiency gradually decreased (more than 10%) after two weeks. The decrement in efficiency might be attributed to the degradation of the antibody immobilized on the electrode surface which could be alleviated by storing in an appropriate package and dry environment.

### Accuracy and precision

**Figure 5:**
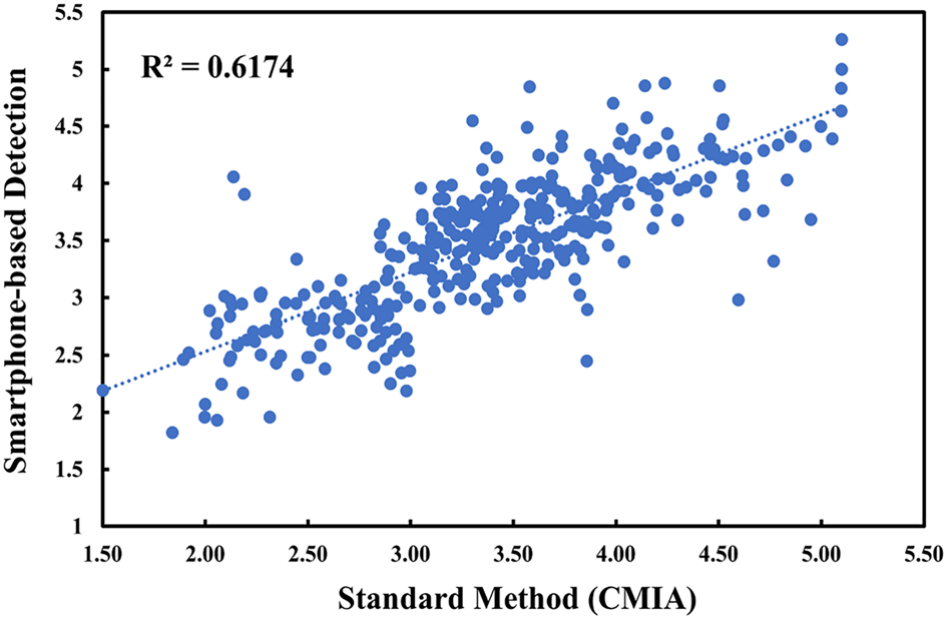
Correlation between HBsAg logarithmic titers obtained from NFC smartphone-based detection and Chemiluminescence microparticle immunoassay (CMIA).

To ensure the accuracy of the proposed HBsAg immunosensor, chemiluminescence microparticle immunoassay (CMIA)^42^ was used as a comparison. The correlation between HBsAg titers of 400 positive samples obtained from our approach and standard method was then established. As illustrated in Fig. 5, a linear relationship was observed with a coefficient of determination (R^2^) value of 0.6174, indicating an insignificant difference between the two methods^43,44^. Additionally, a *t-*test comparison (Table S2) revealed no significant difference between two methods at 95% confidence (*t*-value = 0.63, < *t-*critical = 1.97, α = 0.05), suggesting good agreement between the two methods^45^. Further, the electrode-to-electrode reproducibility (Fig. S6) was evaluated in terms of relative standard deviations (RSDs). The repetitive measurements (n = 10) of three HBsAg concentrations (10 IU/mL, 100 IU/mL, and 1000 IU/mL) provided an acceptable %RSDs of 1.38–2.38% (less than 5%). These results exhibited excellent accuracy and precision of the proposed system.

### Diagnostic testing of HBsAg in clinical samples

To illustrate the capability of this system, the *e*Card sensor combined with the NFC smartphone-based detection system was used for targeting 500 chronic HBV-infected serum samples obtained from King Chulalongkorn Memorial Hospital. The results obtained from our system were validated to those determined by CMIA standard method. The data in Table 1 show that the two approaches have excellent agreement, with sensitivity and specificity of 97.75% and 93%, respectively, demonstrating their success when applied to real patient samples. However, future work will further aim to investigate and integrate the signal enhancement using this smartphone-based technology to meet the need of significant progress in HBV diagnosis.

**Table 1:** The comparison of obtained results between the smartphone-based immunosensor and CMIA method.

		CMIA	
		+	−
Smartphone-based detection	+	391	7
	–	9	93

+ : Positive result, − : Negative result.

## Conclusions

We demonstrated an innovative *e*Card sensor utilizing smartphone-enabled detection system for real-time HBsAg diagnosis. The developed *e*Card sensor was engineered as a portable credit card-sized potentiostat controlled by a wireless NFC network connection of smartphone. Chitosan-supported immobilization matrices were successfully used for simple but effective, reproducible, and stable antibody loading of electrode. The HBsAg quantification was achieved by a simple label-free immunoassay with remarkable sensitivity. The monoclonal antibody readily immobilized to the disposable SPCE exhibited excellent specificity for targeting HBsAg. By applying 500 clinical samples, our biosensing platform shows good agreement compared to the CMIA standard method. As demonstrated, the proposed existing platform offers promise for the simple and rapid detection of HBsAg in a field setting. Furthermore, the adaptation of our approach (by redesigning the specific capture antibody) would allow the targeting of a wide range of medical testing that could be served for deployment in resource-limited settings. This adaptability will allow our NFC sensing technology to be used against many different targets moving forward.

## Materials and methods

### Chemicals

All chemicals employed in this work were analytical grade. Chitosan and glutaraldehyde were obtained from Sigma-Aldrich (USA). Anti-hepatitis B virus surface antigen monoclonal antibody (HBsAg mAb, ab8636) was purchased from Abcam (United Kingdom). BlockPro™ blocking buffer was purchased from Visual Protein (Taiwan). Phosphate buffer saline (PBS) tablet pH 7.4, potassium ferricyanide and potassium ferrocyanide were obtained from Sigma-Aldrich (USA). Milli-Q purified water (R ≥ 18.2 MΩ cm^−1^ at 25 °C) from Merck Millipore was used throughout the experiments. Hepatitis B virus e antigen (HBeAg, ab91273), hepatitis C virus core antigen (HCV-cAg, ab49015), HCV-E2 protein (ab214832), and HCV-NS5a protein (ab180321) used to test cross-reactivity were purchased from Abcam (United Kingdom).

### Apparatus and instruments

All experiments were carried out using [Fe(CN)_6_]^3−/4−^ redox solution in three-electrode cell. A disposable SPCE (MTE-100) was obtained from PICS. Corp. (Taiwan) and the electrode design was shown in Fig. S7. Conductive silver (Ag) ink was utilized as the pseudo-reference electrode (RE). Carbon ink was employed to prepare the working (3 mm i.d.) (WE), counter (CE) electrodes, and conductive pads. The electrochemical characterization including cyclic voltammetry (CV) and electrochemical impedance spectroscopy (EIS) were performed using PalmSens 4 potentiostat (PalmSens Bv, Netherlands). EIS was conducted in the frequency range of 100 kHz to 0.01 Hz and AC potential of 0.25 V. CV was performed from − 0.3 to 0.6 V with scan rate of 0.1 V/s. The amperometric measurements were carried out using the *e*Card sensor (Silicon Craft Technology PLC, Thailand) operated by smartphone (Motorola One) with Android system.

### Design and operation of eCard sensor

A portable *e*Card sensor is designed in the typical credit card size with a dimension of 5.5 cm × 8.5 cm as shown in Fig. S8A. The built-in circuits, consisting of a planar copper antenna, NFC chip (SIC4341) and electrode connector, were created to fit on the printed circuit board (PCB) as presented as the diagram in Fig. S8B. The operating system was controlled by a smartphone via a Chemister Android application. The electrochemical methods and measurement parameters can be directly commanded by the app interface. During the operation procedure, the NFC tag sensor gained the energy emitted from magnetic field of smartphone through the antenna loop. The analog-to-digital data was then converted, and the results were displayed on the screen using real-time measurement data.

### Immobilization of HBsAg Ab

**Figure 6:**
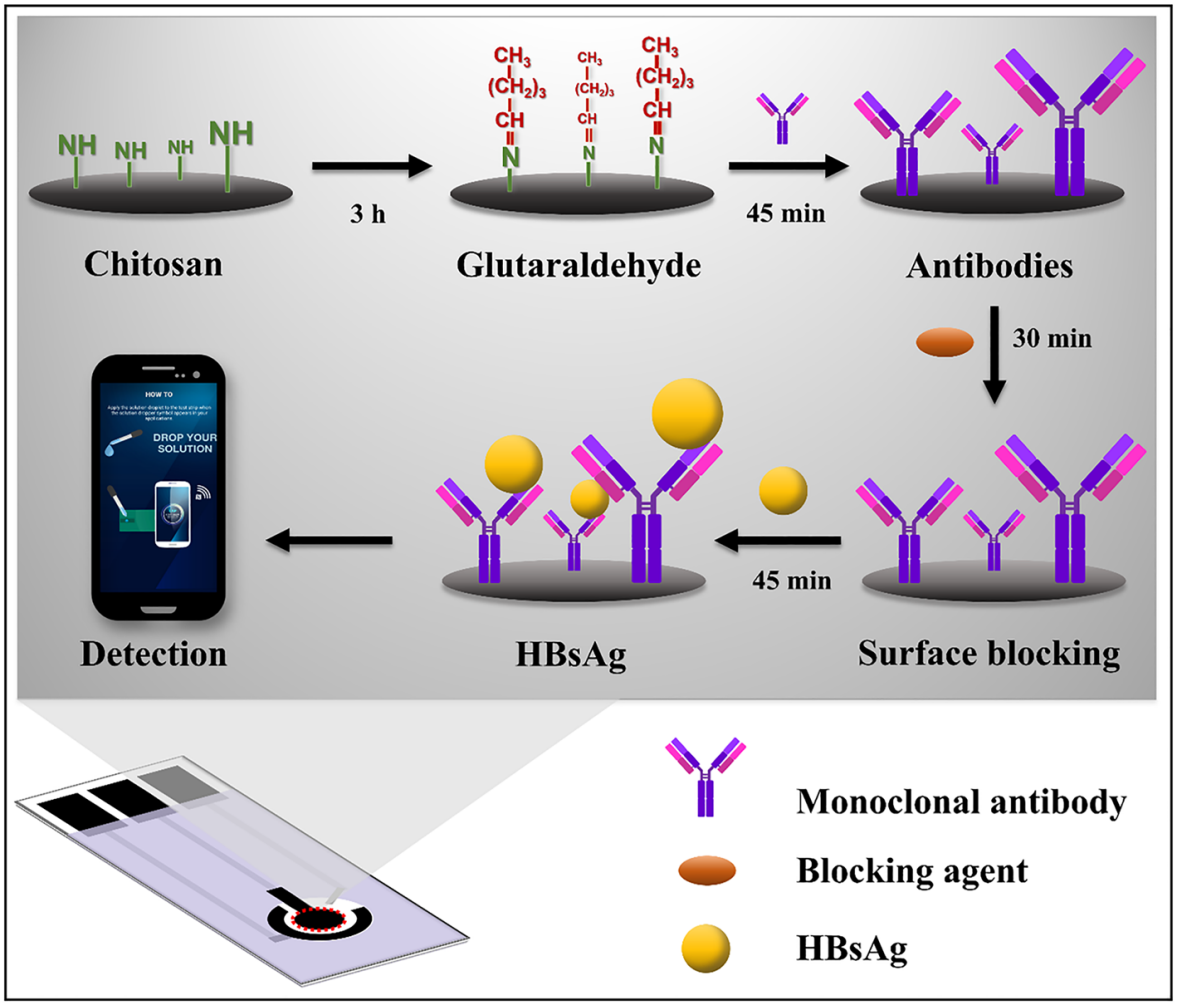
Schematic illustration of stepwise electrode modification for HBsAg electrochemical immunosensor.

To construct the HBsAg immunosensor as shown in Fig. 6, initially, 5 µL of 0.1% (w/v) CHT solution prepared in 1% acetic acid was dropped onto the WE surface and allowed to dry at room temperature. The chitosan modified electrode (CHT/SPCE) was subsequently incubated with 1% (v/v) glutaraldehyde aqueous solution (3 µL) at room temperature for 3 h, followed by washing with PBS (pH 7.4). Next, 2 µL of the anti-HBsAg monoclonal antibody (Ab) was immobilized onto the G/CHT/SPCE modified electrode and incubated at room temperature for 45 min. The Ab/G/CHT/SPCE was then washed with PBS (pH 7.4) to remove the excess and non-immobilized Ab. Following, the surface blocking of non-specifically adsorbed proteins was done by adding 2 µL of BlockPro™ (BP) solution to the Ab/G/CHT/SPCE-modified electrodes, incubating for 30 min and washing with PBS (pH 7.4) to obtain the BP/Ab/G/CHT/SPCE. The prepared HBsAg immunosensor can be stored for a long term stability (shelf life) at 4 °C prior to use.

### Smartphone-based amperometric detection using eCard sensor

Prior to electrochemical measurement, a prepared serum sample (2 µL) was dropped onto the working electrode surface and incubated for 45 min. The unbound protein on the electrode surface was then washed by PBS (pH 7.4). The prepared sensor was inserted into the electrode connector. The wireless connection between a smartphone and *e*Card sensor was operated by the Chemister app. After selecting the electrochemical method and setting all parameters, the connection allows starting by tapping a smartphone onto the tapping area of *e*Card sensor. Once the connection was established, 60 µL of 2 mM [Fe(CN)_6_]^3−/4−^ was introduced onto the electrode surface and the detection system began to collect data. The real-time results were displayed on the smartphone app. All application windows for the operation processes are shown in Fig. S9.

### Real samples preparation

The clinical serum samples including 400 HBsAg positive and 100 HBsAg negative were obtained from patients who attended King Chulalongkorn Memorial Hospital, Bangkok, Thailand. This study was performed in accordance with the Declaration of Helsinki for the participation of human individuals. The written inform consents were obtained from all participants involved in the study. All experimental protocols were approved by the Institute Ethics Committee of Faculty of Medicine, Chulalongkorn University (IRB. No. 0067/65). For the preparation of serum, briefly, the whole blood samples were collected in the red topped tubes and then the clot was removed by centrifugation at 1500 × g for 15 min, resulting in a supernatant and liquid component (serum). All designated serum samples were stored at − 80 °C prior to electrochemical measurement using the aforementioned procedure. Finally, the obtained results were validated with those obtained from the standard chemiluminescence microparticle immunoassay (CMIA).

## Supplementary Information

Supplementary Information.

## Data Availability

The datasets used and/or analyzed during the current study available from the corresponding author on reasonable request.
