# Supplementary material for: An innovative wireless electrochemical card sensor for field-deployable diagnostics of Hepatitis B surface antigen
Source: Sci Rep. 2023 Mar 2;13:3523. doi: 10.1038/s41598-023-30340-5 (PMC9981757; doi:10.1038/s41598-023-30340-5)
Supplement: Supplementary file 1 — Supplementary Information. [file 41598_2023_30340_MOESM1_ESM.docx]

**Supporting Information (SI)**

**An Innovative Wireless Electrochemical Card Sensor for Field-deployable Diagnostics of Hepatitis B surface Antigen**

Prinjaporn Teengam^a^, Pisit Tangkijvanich^b^, Natthaya Chuaypen^b,*^ and Orawon Chailapakul^a,*^

*^a^Electrochemistry and Optical Spectroscopy Center of Excellence, Department of Chemistry, Faculty of Science, Chulalongkorn University, Pathumwan, Bangkok, 10330, Thailand*

*^b^Center of Excellence in Hepatitis and Liver Cancer, Department of Biochemistry, Faculty of Medicine, Chulalongkorn University, Pathumwan, Bangkok, 10330, Thailand*

*Corresponding authors at: E-mail address:

Orawon Chailapakul, corawon@chula.ac.th; Natthaya Chuaypen, natthaya.ch56@gmail.com


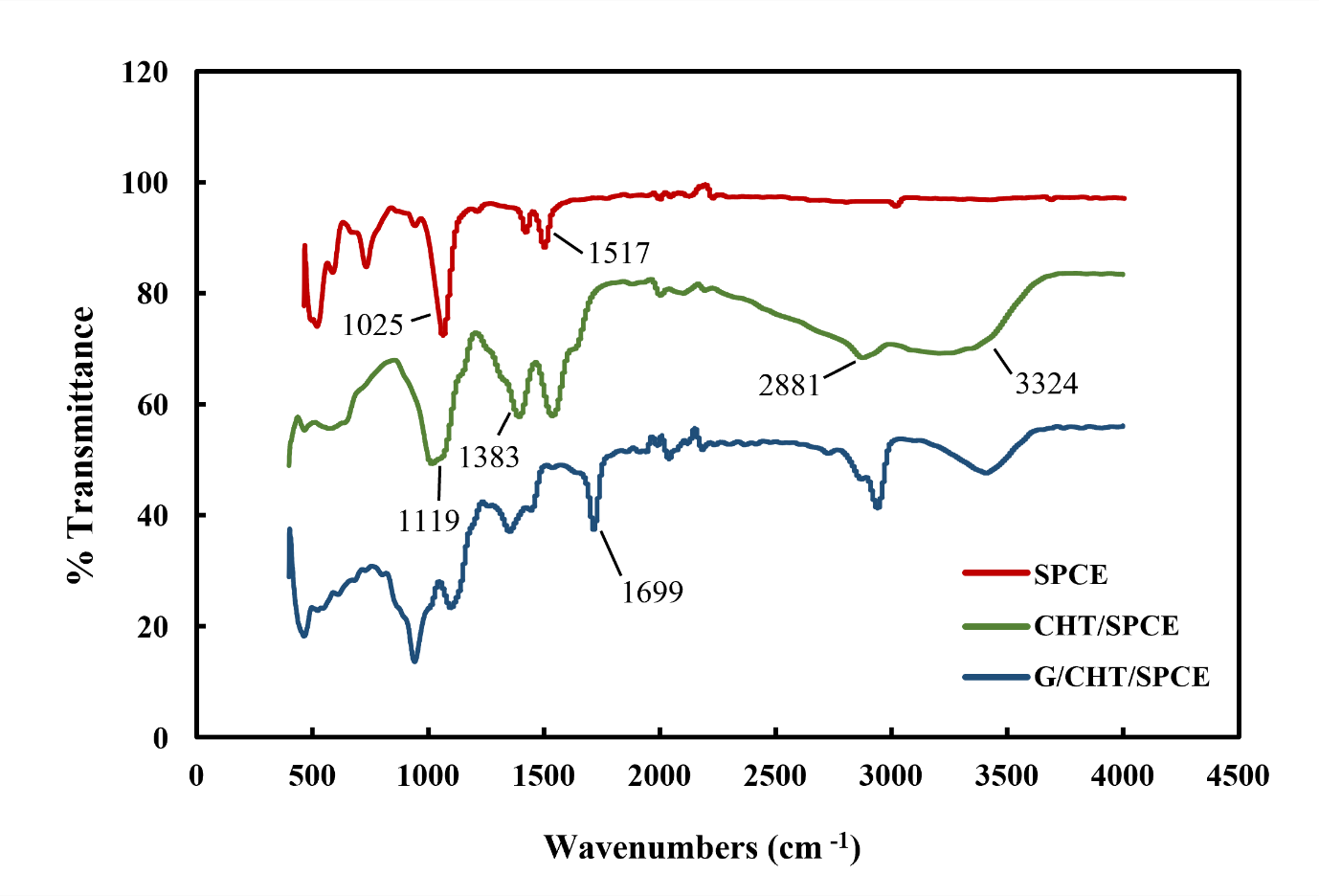


**Fig. S1** The comparison of ATR-FTIR spectra of SPCE, CHT/SPCE and G/CHT/SPCE.


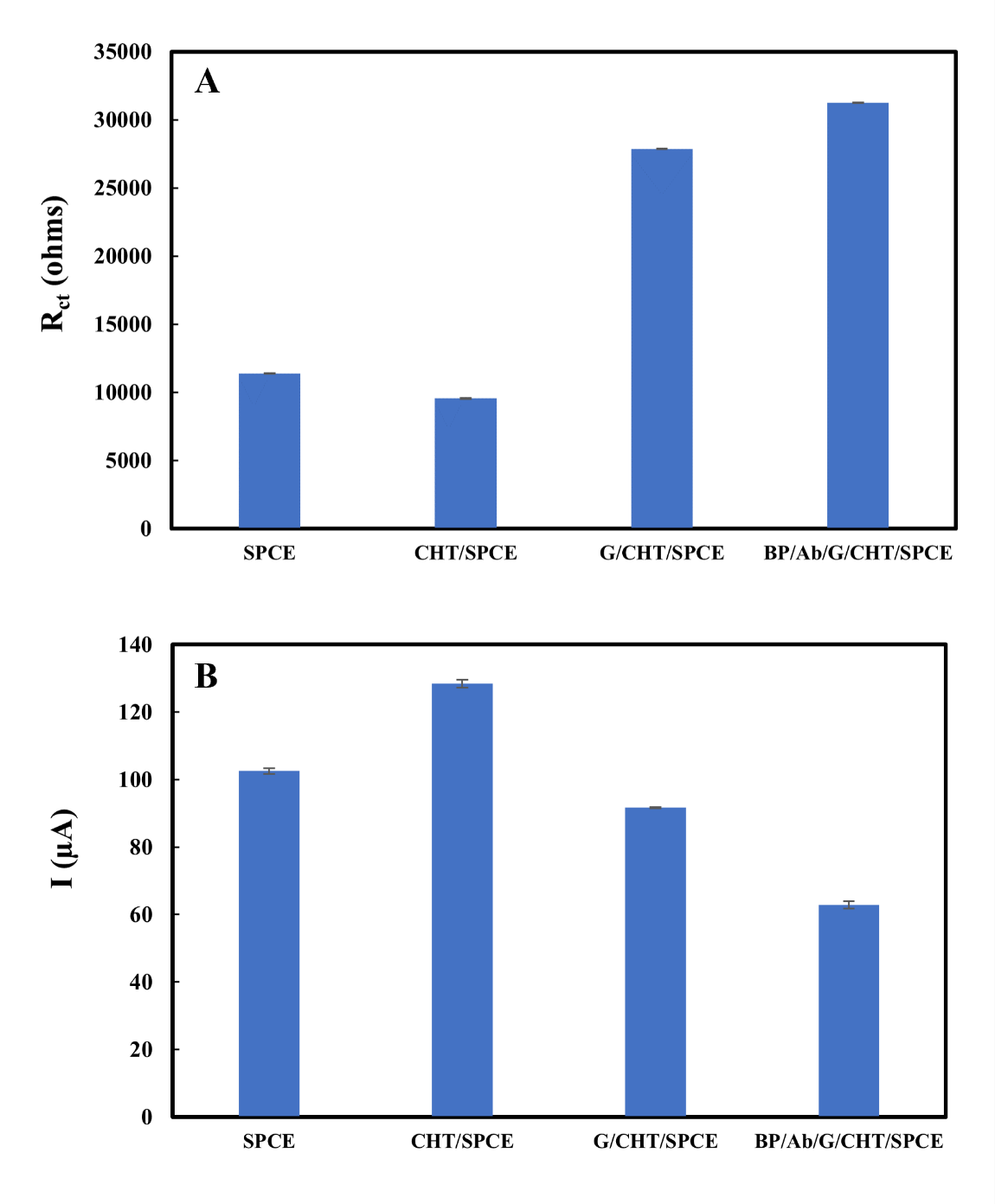


**Fig. S2** (A) The R_ct_ and (B) the current signal obtained in a stepwise electrode modification using 5 mM [Fe(CN)_6_]^3-/4-^.


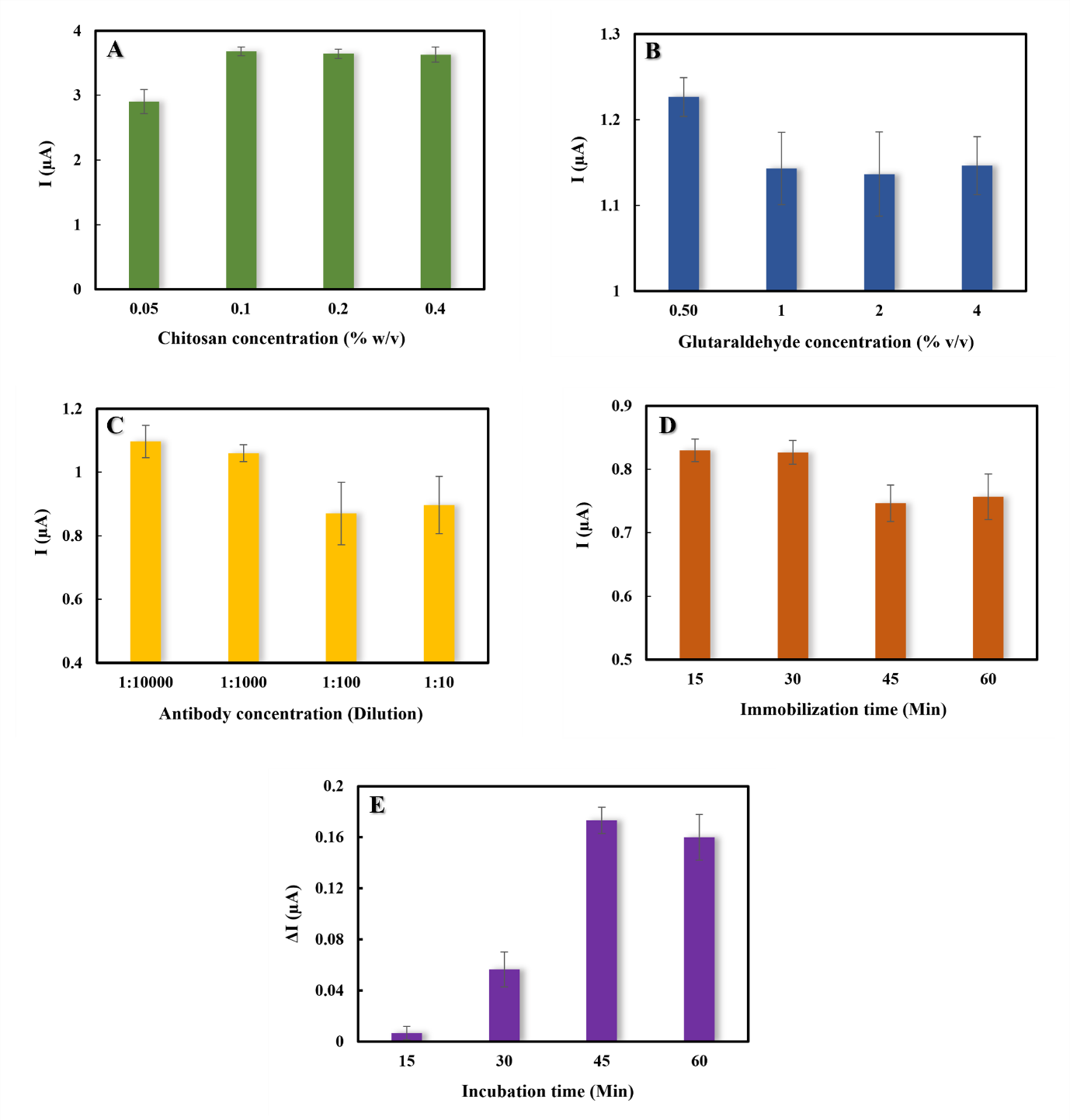


**Fig. S3** The influence of (A) chitosan concentration obtained by serial dilution, (B) Glutaraldehyde concentration, (c) Antibody concentration, (D) Immobilization time and (E) Incubation time.


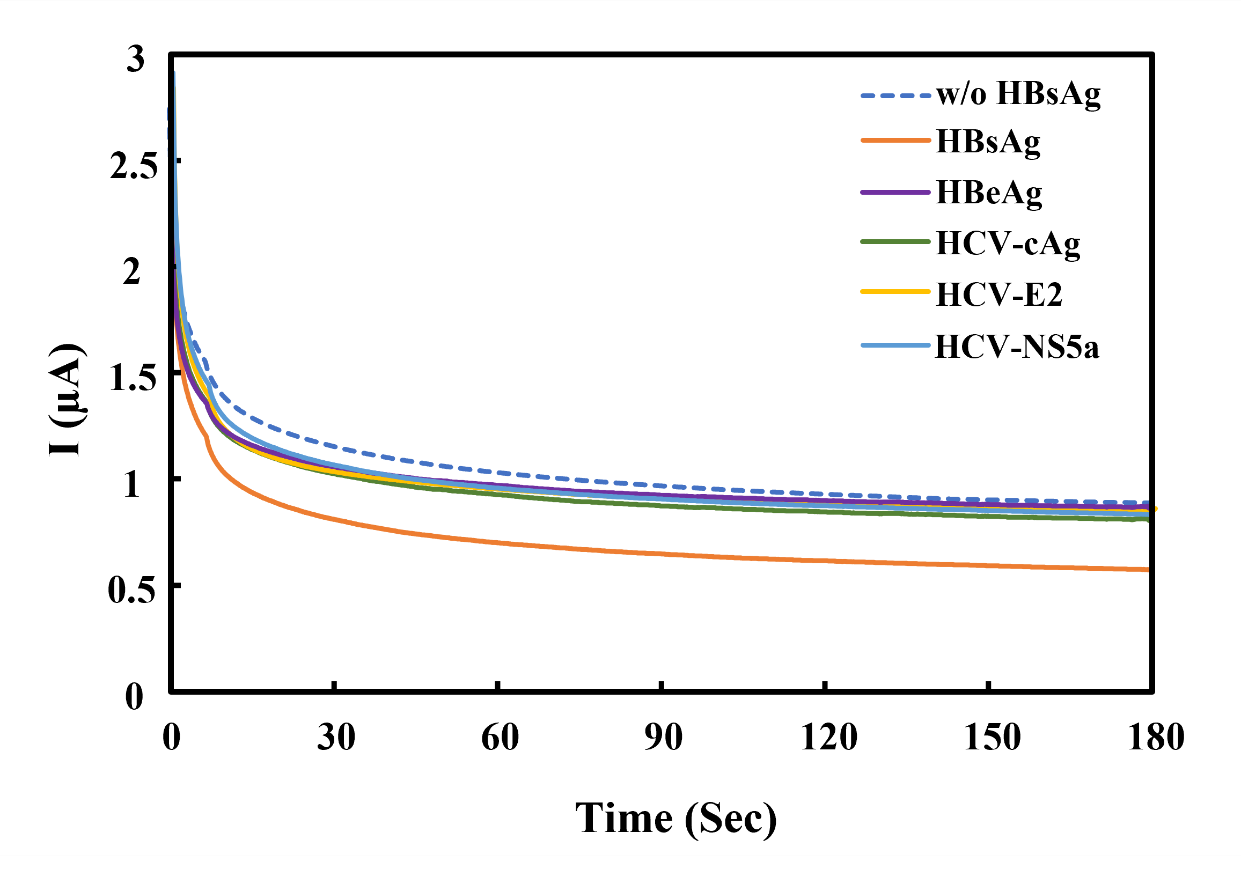


**Fig. S4** The ampermetric curve derived from the proposed sensor after addition of HBsAg and various virus antigens.


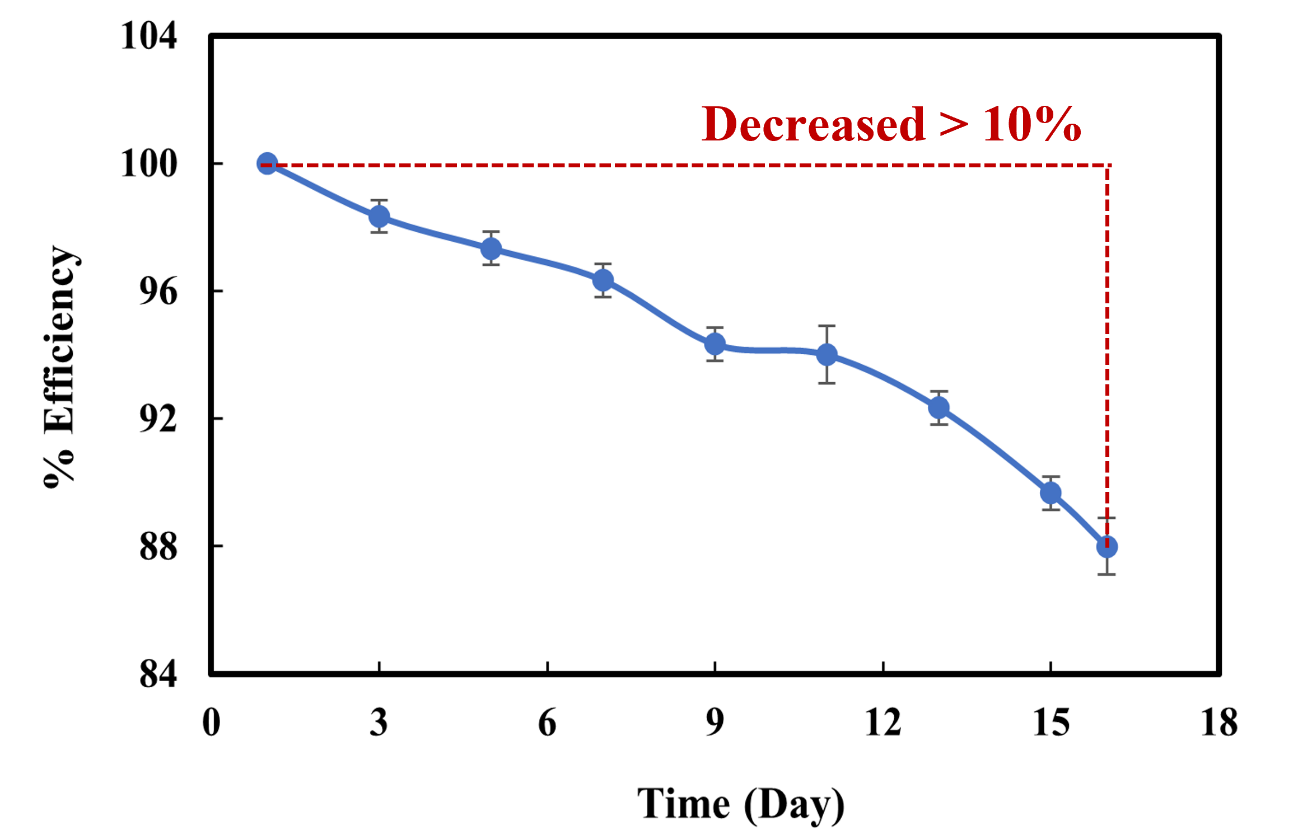


**Fig. S5** The storage stability of the HBV electrochemical immunosensor.


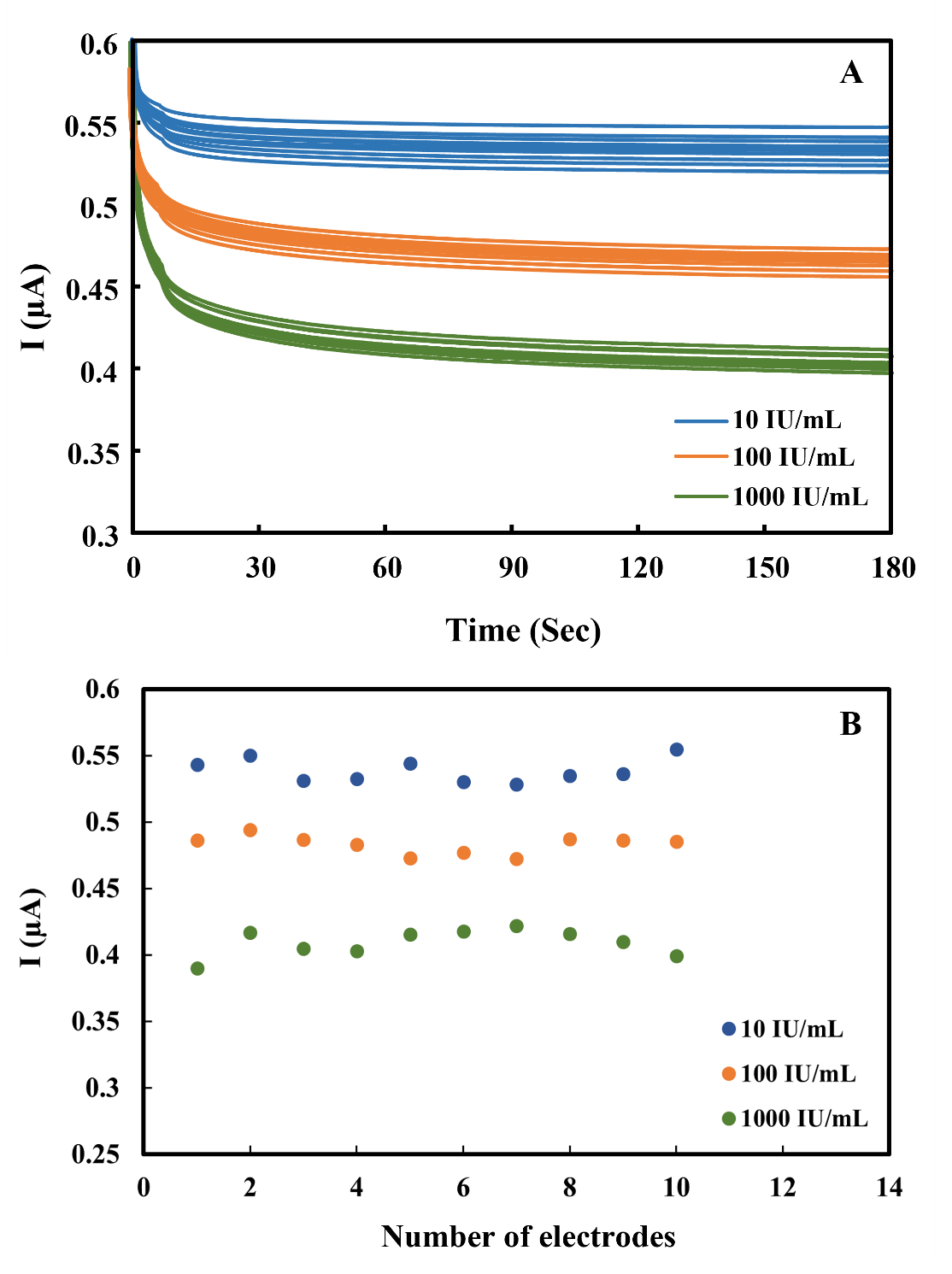


**Fig. S6** (A) The amperometric curve and (B) the current response of ten HBsAg electrochemical immunosensors.


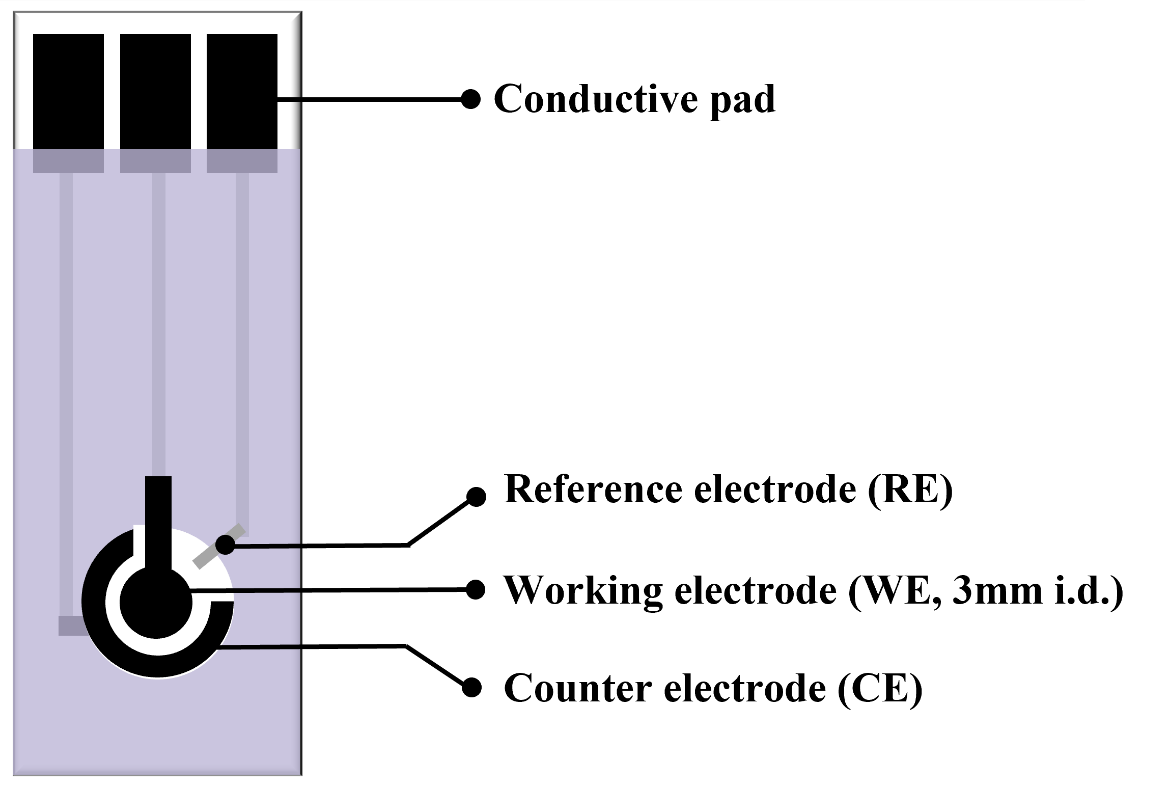


**Fig. S7** The design of disposable electrode (MTE-100).


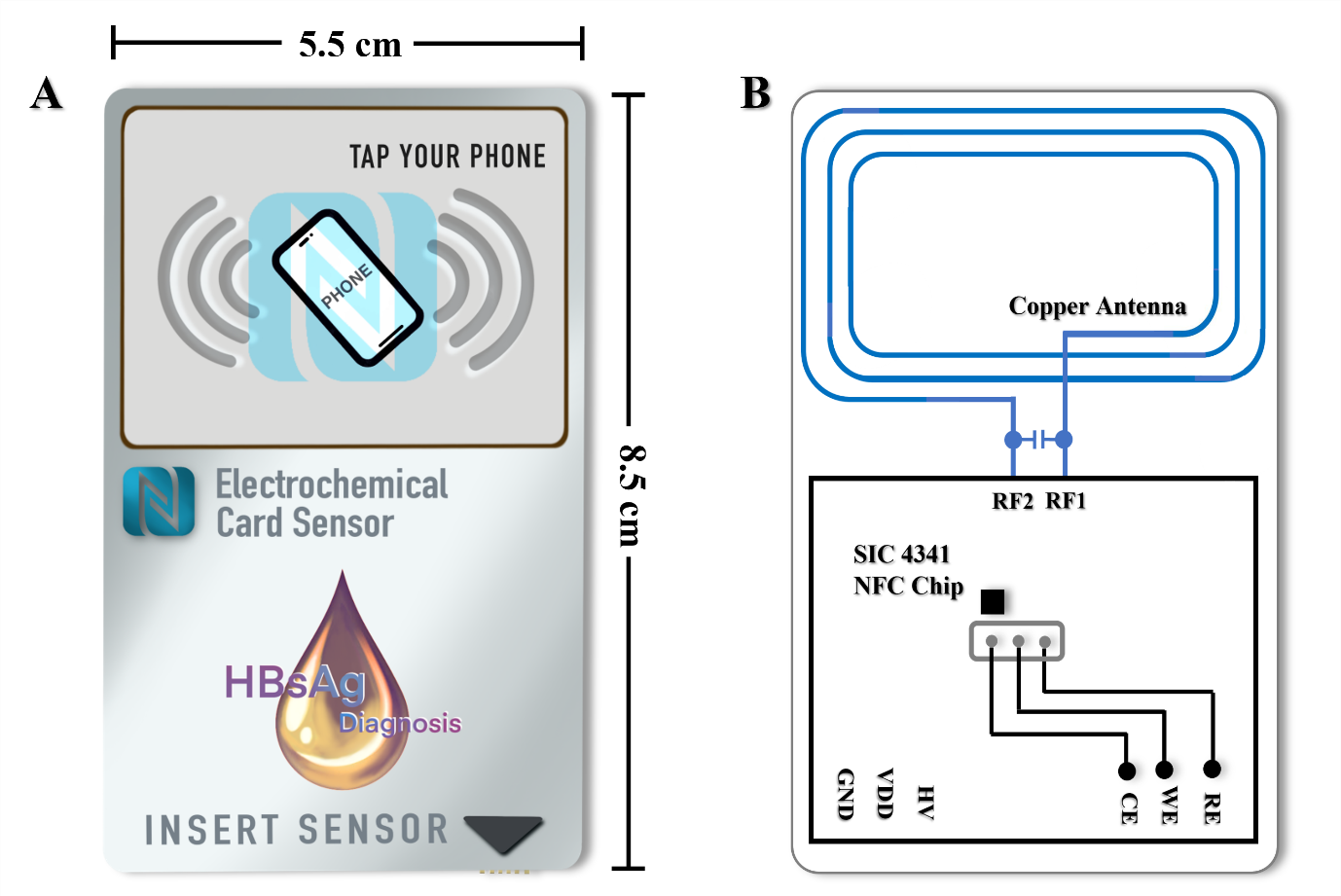


**Fig. S8** (A) The design of electrochemical card (*e*Card) sensor and (B) the diagram of the printed circuit board (PCB).


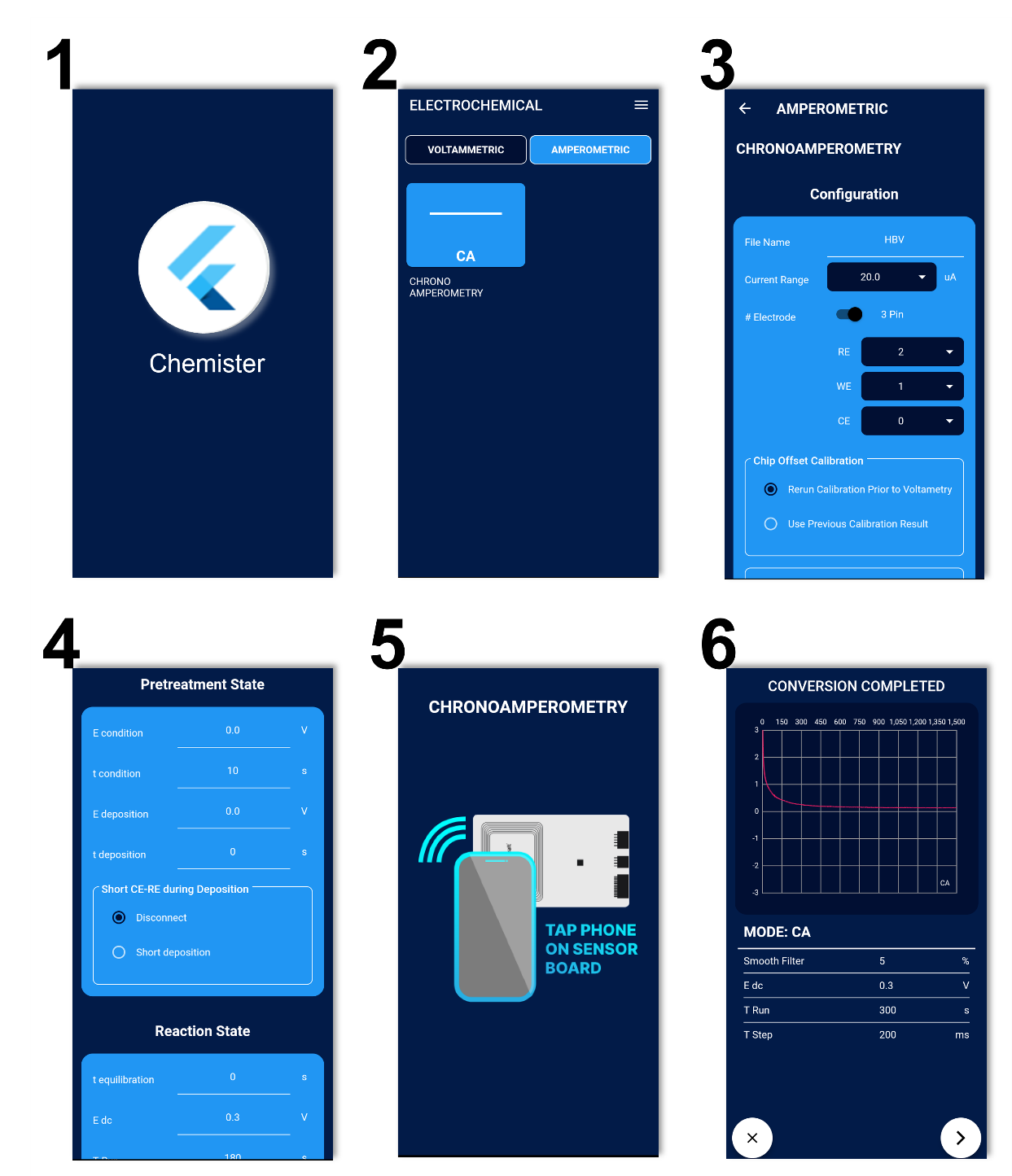


**Fig. S9** The windows of Chemister Application on a smartphone for all operational processes.

**Table S1.** The analytical performance of various electrochemical immunosensor for HBsAg detection

| Electrochemical Technique | Detection Assay | Detection limit | Portability | Ref |
| --- | --- | --- | --- | --- |
| Amperometry | Sandwich-type immunoassay | 166 fg/mL | No | [[1](#_ENREF_1)] |
| DPV | Sandwich-type immunoassay | 6.7 fg/mL | No | [[2](#_ENREF_2)] |
| DPV | Label-free immunoassay | 10.2 fg/mL | No | [[3](#_ENREF_3)] |
| DPV | Sandwich-type immunoassay | 3x10^-11^ µg/mL | No | [[4](#_ENREF_4)] |
| Amperometry | Label-free immunoassay | 9.55 IU/mL | Yes | This work |

Abbreviation: DPV, Differential Pulse Voltammetry

**Table S2.** *t*-Test: Paired Two Sample for Means

|  | **Variable 1** | **Variable 2** |
| --- | --- | --- |
| **Mean** | **3.382657105** | **3.36255268** |
| **Variance** | **0.659053651** | **0.509981685** |
| **Observations** | **400** | **400** |
| **Pearson Correlation** | **0.785766315** |  |
| **Hypothesized Mean Difference** | **0** |  |
| **df** | **399** |  |
| **t Stat** | **0.630828231** |  |
| **P(T<=t) one-tail** | **0.264257003** |  |
| **t Critical one-tail** | **1.648681534** |  |
| **P(T<=t) two-tail** | **0.528514005** |  |
| **t Critical two-tail** | **1.965927296** |  |

**References**

[1] F. Pei, P. Wang, E. Ma, Q. Yang, H. Yu, C. Gao, et al., A sandwich-type electrochemical immunosensor based on RhPt NDs/NH2-GS and Au NPs/PPy NS for quantitative detection hepatitis B surface antigen, Bioelectrochemistry, 126(2019) 92-8.

[2] M. Li, P. Wang, F. Pei, H. Yu, P. Chen, Y. Dong, et al., Highly sensitive immunosensor for Hepatitis B surface antigen detection based on a novel signal amplification system of gold nanorods and mesoporous Au@Pd@Pt core-shell nanospheres, Journal of Electroanalytical Chemistry, 809(2018) 14-21.

[3] Z. Tan, H. Dong, Q. Liu, H. Liu, P. Zhao, P. Wang, et al., A label-free immunosensor based on PtPd NCs@MoS2 nanoenzymes for hepatitis B surface antigen detection, Biosensors and Bioelectronics, 142(2019) 111556.

[4] R. Hallaj, M. Mottaghi, Z. Ghafary, F. Jalali, Ultrasensitive electrochemical detection of hepatitis b virus surface antigen based on hybrid nanomaterials, Microchemical Journal, 182(2022) 107958.
